# The flexible dialyser membrane – a source of error in plasma creatinine determination

**DOI:** 10.1155/S1463924680000594

**Published:** 1980

**Authors:** Trevor A. Walmsley, Richard T. Fowler, Maxwell H. Abernethy, Michael Lever, David J. Munster

**Affiliations:** Department of Clinical Biochemistry Christchurch Hospital Christchurch New Zealand


### The flexible dialyser membrane – a source of error in plasma creatinine determination

**Trevor A. Walmsley, Richard T. Fowler, Maxwell H. Abernethy, Michael Lever and David J. Munster**
*Department of Clinical Biochemistry, Christchurch Hospital, Christchurch, New Zealand.*

### Introduction

Dialysis is frequently used in continuous flow analysis to separate low molecular weight analytes from protein containing samples. The dialyser assembly consists of two clear plastic blocks with matching donor and recipient grooves, between which is sandwiched a semipermeable membrane. The air-segmented donor (diluted sample) stream and recipient streams flow at constant rates along the grooves. This allows partial diffusion of the analyte across the membrane. The analyte is transferred into the recipient stream. On leaving the dialyser, reagents are added to this stream in order to produce a response to the analyte and to provide a quantitative measurement. Detergent is often added to the sample stream to facilitate the analysis.

At the detergent concentrations commonly used in continuous flow assays the pressure within the manifold drastically increases when a protein containing sample is analysed [1]. The cellophane membranes used are thin and flexible, therefore changes in the pressure across a dialyser membrane affects the conformation of the membrane. The relative volumes and flow rates of the donor and recipient streams within the dialyser are also changed. This phenomenon was investigated particularly for a plasma creatinine assay commonly used in clinical chemistry [2]. Figure 1 shows a schematic flow diagram of the system used. In the assay, the high baseline absorbance of the picric acid reagent is sensitive to momentary changes in flow rate of the recipient stream from the dialyser.

### Materials and methods

All chemicals used were Fisons "Analytical Reagent" grade (Loughborough, U.K.).

The continuous flow equipment and consumables used were purchased from Technicon Instrument Corporation, Tarrytown, New York, USA. Type C dialyser membranes were used throughout this work.

Pressure measurements were made on both donor and recipient streams at points just prior to air-segmentation and level with the dialyser membrane. A simple water manometer was used for the measurements. Unless otherwise specified, the waste streams from the colorimeter and dialyser were open to the atmosphere at a point 175 mm below the level of the dialyser membrane. Pressure differences across the dialyser membrane were created by raising or lowering the waste stream from the dialyser.

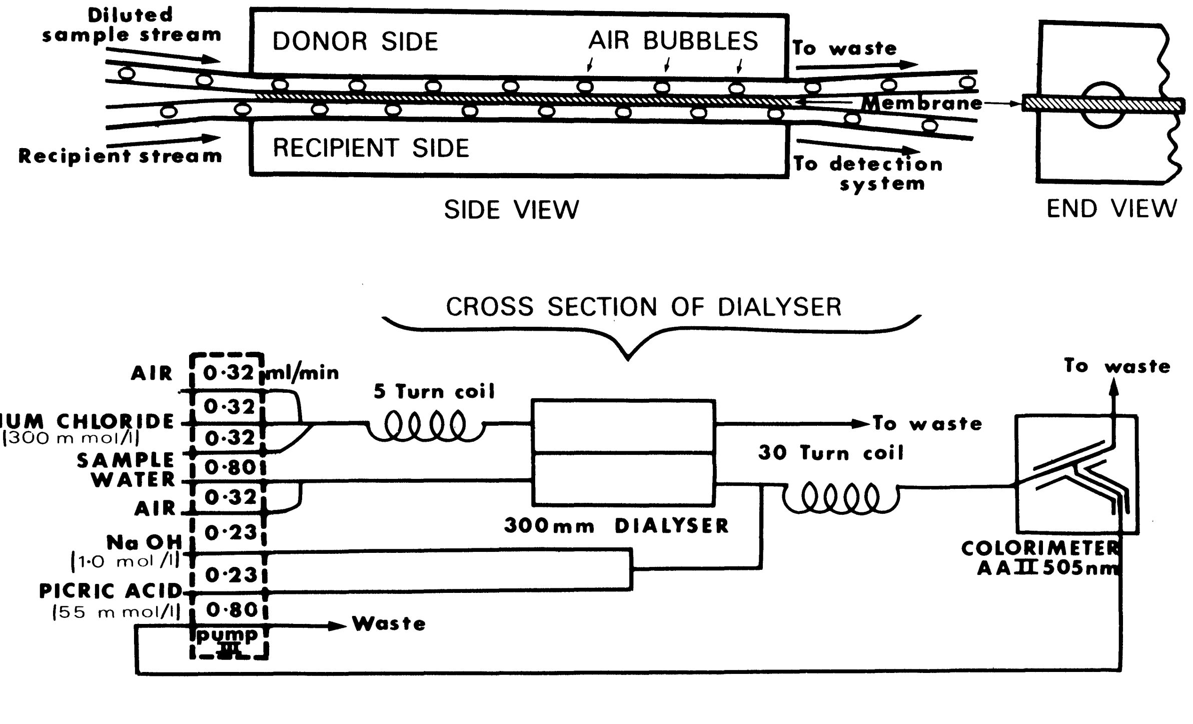

*Figure 1. Manifold diagram of the plasma creatinine analyser (inset contains details of the construction of the dialyser).*

Variations in the donor and recipient volumes within the dialyser were calculated from the transit times of segments through the recipient compartment of the dialyser. Care was taken to ensure that the dialyser membrane was tight and free of wrinkles. The total volume of the 300 mm dialyser used was 863 $\mu$l.

### Results

### Changes in dialyser volume with pressure

As the pressure on the donor side of the dialyser was raised from −2.5 kPa relative to the recipient stream, initially there was a small decrease in the volume of the recipient compartment of the dialyser of 2.5 $\mu$l kPa$^{-1}$ (Figure 2). However, as the pressure across the dialyser membrane equalises, any further increase in pressure of the donor stream produces a dramatic shift in the conformation of the dialyser membrane as shown by the large decrease in volume of the recipient stream of the dialyser, also shown in Figure 2. Further increases in pressure cause small decreases in the volume of the recipient compartment of 5 $\mu$l kPa$^{-1}$. Decreasing the pressure on the donor side of the dialyser showed that these changes were reversible and that no hysteresis was evident.

### Changes in membrane conformation when 0.3 g/l Brij 35 is added to the sample diluent reagent (300 mmol/l sodum chloride)

During the wash phase of routine operation of the plasma creatinine analyser, the pressure on the donor side of the dialyser is lower than during the sample phase. The volume of the recipient compartment of the dialyser is decreased by 56 $\mu$l (Figure 2, point B) as the membrane alters conformation. The flow rate of the recipient stream emerging from the dialyser momentarily increases as the volume of the recipient stream decreases. This results in extra dilution of the picric acid solution which results in a dip in the baseline before the first peak emerges (Figure 3). Conversely, as water is again aspirated at the end of a run of specimens, the pressure on the donor side of the dialyser decreases and the flow rate of the recipient stream from the dialyser momentarily slows down. This results in underdilution of the picric acid solution causing an increase in height of the last peak (Figure 3a).

A commercial control serum of comparable creatinine concentration to the pooled plasma sample was also analysed. The increase in volume of the recipient stream of the dialyser was only 3 $\mu$l and no distorted peaks were observed (Figure 3b).

The addition of a detergent to the control serum during the manufacturing process may explain the difference in behaviour compared with the pooled plasma sample [1].

### Changes in membrane conformation when 30 g/l Brij 35 is added to the sample diluent reagent

When 30 g/l Brij 35 was added to the sample diluent reagent, the pressure of the donor side of the dialyser is lowered, but remains outside the critical region where small changes in pressure cause a large change in membrane conformation as shown as point C in Figure 2. However, the major advantage of using this detergent concentration is that no change in pressure occurs when a plasma sample is aspirated into the creatinine analyser [1] (Figure 2, point C). The conformation of the membrane remains constant and the peak shapes for plasma samples appear like those in Figure 3b.

### Discussion

Many workers consider the dialyser membrane to be an inert rigid sieve used for deproteinisation on continuous flow manifolds. In fact the dialyser membrane is flexible and can significantly change its conformation during the course of an analysis. The change in conformation when the pressure difference across the membrane is large demonstrates its elasticity, whereas the dramatic changes that occur when there is no substantial pressure difference across the membrane reflects its slackness. Both these factors are inherent properties of the dialyser membrane. The degree of slackness in the dialyser membrane would be operator-dependent. This work underlines the necessity for a tight, wrinkle-free dialyser membrane to minimise sudden large changes in membrane conformation.

At the suboptimal detergent concentrations normally recommended for the analysis of plasma creatinine, the

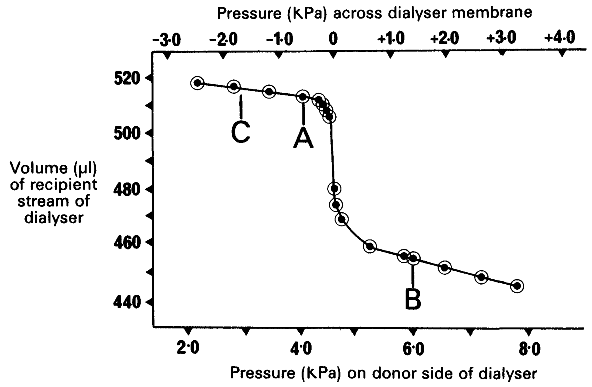

*Figure 2. Change in volume of recipient stream of dialyser with pressure. The pressure of the donor stream of the dialyser was varied by raising or lowering the waste stream from the dialyser. (A) Pressure under normal operation using a sample of water and a diluent containing 0.3 g/l Brij 35. (B) Pressure under normal operation using a sample of plasma and a diluent containing 0.3 g/l Brij 35. (C) Pressure under normal operation using either a sample of water or plasma and a diluent containing 30 g/l Brij 35. (The total volume of the dialyser (donor plus recipient compartments) = 863 µl.*

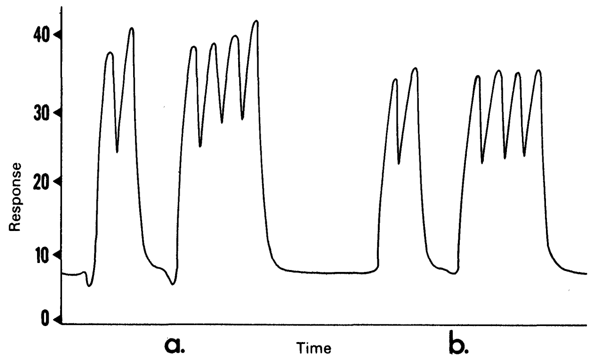

*Figure 3. (a) Peak shapes given by pooled plasma samples. Creatinine value 0.68 mmol/l. A decrease in the volume of the recipient compartment of 56 µl was observed on sampling the plasma. (b) Peaks given by a quality control serum of comparable creatinine level. A decrease in the volume of the recipient compartment of 3 µl was observed on sampling the quality control serum.*

dialyser membrane would continuously flex as samples of different protein concentrations are analysed – the peak height of a specimen does not necessarily reflect its creatinine level. In the authors' experience this produces many bizarre peak shapes within every batch and the last peak is always higher than expected. One way of minimising conformational changes in the dialyser membrane is to raise the waste donor stream of the dialyser. However, under these conditions small changes in conformation still occur due to the elasticity in the membrane, and the last peak in every batch is still higher than expected. A satisfactory solution to this problem has been found by minimising pressure differences between samples. A plasma creatinine analyser has been operated for the last 18 months using a sample diluent of sodium chloride (300 mmol/l) and 10 g/l of Brij 35. Over 75,000 specimens were analysed during this time. A higher concentration of 30 g/l is not used, in order to reduce the possibility of phase separation when samples of high total protein are analysed [1].

ACKNOWLEDGEMENT
The authors wish to acknowledge the financial support from the Medical Research Council of New Zealand and constructive discussions with Dr C. M. André.

REFERENCES
[1] Walmsley, T. A., *Clin. Chim. Acta*, 1979, **99**, 53-57.
[2] Technicon Method No. AAII-11, 1970, Technicon Instrument Corporation, Tarrytown, New York.

### A bichromatic method for the enzymatic determination of serum triglycerides with the Gemsaec Fast analyser

**P. Bijster*, H. L. Vader and C. L. J. Vink**
*Clinical Laboratory, St. Joseph Hospital, Aalsterweg 259, Eindhoven (The Netherlands).*

### Introduction

Fully enzymatic methods for the determination of serum triglycerides may be carried out in either a kinetic or an equilibrium mode. Both kinetic and equilibrium methods have been adapted for use on centrifugal analysers. Kinetic (or fixed-time rate) analyses are carried out by adapting the reaction conditions to achieve first or pseudo-first-order kinetics [1-5]. Equilibrium analyses on centrifgual analysers deal with some special problems concerning the initial absorbance reading [1,6]. If a slight lag-phase is present in the first stage of the reaction some analysers can read an initial absorbance during this time. Otherwise 'zero-time' extrapolation procedures must be used despite the difficulties these present. Another drawback of equilibrium analyses, especially for centrifugal analysers, is the long reaction time.

A fully enzymatic equilibrium method adapted to the Gemsaec Fast analyser which takes less than two minutes of instrument time is described. It is a modification of the method described by Bucolo *et al* [7]. In this procedure the triglycerides are released and hydrolysed by a mixture of lipase and α-chymotrypsin. Subsequently, the released glycerol is converted to glycerol-1-phosphate in the presence of glycerol kinase and ATP. Phosphoenol pyruvate, included in the reagent, is converted by pyruvatekinase to pyruvate resulting in the regeneration ATP from ADP. The reduction of pyruvate to lactate in the presence of lactate dehydrogenase with concurrent oxidation of $NADH_2$ to $NAD^+$ is used as the indicator reaction. Both the hydrolysis and the elimination of glycerol are carried out at 37°C in the transfer disc outside the analyser within 10 minutes.

To minimise optical interferences, the absorbance of the reaction mixture is read after 10 minutes at two wavelengths, namely 340 nm and 380 nm. There is no optical interference by lipemic sera as the absorbances are determined after complete hydrolysis of the lipids. The concentration of triglycerides is calculated by subtracting the absorbance differences (A 340 nm – A 380 nm) of the reagent blank and determinations compared to that of a glycerol standard.

*Correspondence to this author

### Materials

### Eskalab triglyceride reagent

Eskalab substrate vials (89804) and Eskalab glycerol kinase vials (89802), obtained from Smith Kline Instruments, Inc., Sunnyvale, California 94086, USA, were dissolved in bidistilled water according to the manufacturer's recommendations. The reactants in the reagent mixture have the following approximate concentrations:

| | |
|---|---|
| *Substrate mixture* | |
| Reduced nicotinamide adenine dinucleotide | 0.3 mmol/l |
| Pyruvate kinase | $0.8 \times 10^3$ IU |
| Lactate dehydrogenase | $0.7 \times 10^3$ IU |
| $Mg^{2+}$ | 6 mmol/l |
| Lipase | $300 \times 10^3$ IU |
| α-chymotrypsin | $13 \times 10^3$ IU |
| Phosphoenol pyruvate | 0.8 mmol/l |
| Bovine serum albumin | 1.7 g/l |
| Adenosine triphosphate | 0.5 mmol/l |
| Buffer: (pH 7.1 ± 0.2) | |
| Potassium dihydrogen phosphate | 10 mmol/l |
| di-Potassium hydrogen phosphate | 63 mmol/l |

*Glycerol kinase suspension*
Glycerol kinase not less than 20 IU/vial.

